# Tauroursodeoxycholic Acid Protects Retinal Pigment Epithelial Cells from Oxidative Injury and Endoplasmic Reticulum Stress In Vitro

**DOI:** 10.3390/biomedicines8090367

**Published:** 2020-09-21

**Authors:** Reem Hasaballah Alhasani, Mohammad Almarhoun, Xinzhi Zhou, James Reilly, Steven Patterson, Zhihong Zeng, Xinhua Shu

**Affiliations:** 1 Department of Biology, Faculty of Applied Science, Umm Al-Qura University, Makkah 24381, Saudi Arabia; rhhasani@uqu.edu.sa; 2 Department of Biological and Biomedical Sciences, Glasgow Caledonian University, Glasgow G4 0BA, UK; MALMAR200@caledonian.ac.uk (M.A.); Xinzhi.Zhou@gcu.ac.uk (X.Z.); J.Reilly@gcu.ac.uk (J.R.); Steven.patterson@gcu.ac.uk (S.P.); 3 College of Biological and Environmental Engineering, Changsha University, Changsha 410022, China; 4 Department of Vision Science, Glasgow Caledonian University, Glasgow G4 0BA, UK; 5 School of Basic Medical Sciences, Shaoyang University, Shaoyang 422000, China

**Keywords:** tauroursodeoxycholic acid, retinal pigment epithelial cell, oxidative stress, endoplasmic reticulum stress, protection

## Abstract

Retinal degeneration is characterized by the dysfunction of retinal cells. Oxidative and endoplasmic reticulum (ER) stress play an important role in the pathogenesis and progression of retinal degeneration. Tauroursodeoxycholic acid (TUDCA) has been demonstrated to have protective effects in in vitro and in vivo retinal degeneration models. To fully understand the molecular mechanisms of TUDCA’s protection, we first treated human retinal pigment epithelial (RPE) cells, ARPE-19, with H_2_O_2_ or H_2_O_2_ plus TUDCA for 24 h. RPE cells co-exposed to TUDCA had higher cell viability and lower cell death rate compared to cells exposed to H_2_O_2_ alone. TUDCA significantly increased antioxidant capacity in H_2_O_2_-treated RPE cells by decreasing the generation of reactive oxygen species (ROS) and Malondialdehyde (MDA), upregulating the expression of antioxidant genes, and increasing the generation of glutathione (GSH). TUDCA also inhibited inflammation in H_2_O_2_-challenged RPE cells by decreasing the expression of proinflammatory cytokines. Furthermore, TUDCA suppressed thapsigargin-induced ER stress in RPE cells, as demonstrated by decreased the expression of CCAAT-enhancer-binding protein homologous protein (CHOP) and apoptosis. Our present study suggests that TUDCA can protect RPE cells against oxidative damage, inflammation, and ER stress and may benefit patients with retinal degeneration.

## 1. Introduction

The retinal pigment epithelium (RPE) is a hexagonal monolayer of highly specialized cells that lie between the neurosensory retina and the choroid. The RPE is responsible for maintaining photoreceptor function by absorbing scattered light, providing nutrition, removing metabolic waste, maintaining the visual cycle, phagocytosing the shed outer segments of photoreceptors, and secreting a range of functional mediators [1]. Due to continuous light absorption, high metabolic activity, and phagocytosis of photo-oxidized outer segments, the RPE has a high level of oxidative stress. The RPE has developed an antioxidant defense system to maintain normal function under physiological conditions. With aging or in disease states, increased oxidative stress causes RPE dysfunction, which contributes to the progression of retinal degeneration, such as that present in age-related macular degeneration (AMD) [2]. Oxidative stress targeted therapy is recommended as one option for treating retinal diseases. 

Tauroursodexycholic acid (TUDCA), a taurine-conjugated bile acid, is presented at a very low level in human bile, but at a high level in bear bile [3]. Bear bile, as traditional Chinese medicine, has been used for over three thousand years to treat a variety of diseases, including visual impairments [4,5]. In the last 15 years, many studies have provided evidence showing the protective effects of TUDCA against retinal degeneration in retinal cell lines and animal models [6]. TUDCA treatment ameliorates photoreceptor death in naturally occurring, knockout, and transgenic rodent models of retinal diseases [3,7,8,9,10,11]. TUDCA also shows the protection of ganglion cells from n-methyl-d-aspartate-induced retinal damage in rats [12] and a rat model involving crushed optic nerve [13]. Recently, we have examined the protective effect of TUDCA against photoreceptor degeneration in a *retinitis pigmentosa GTPase regulator* (*Rpgr*) knockout mouse model of retinitis pigmentosa. We found that TUDCA attenuated caspase-dependent photoreceptor cell death, inhibited retinal microglial activation, and suppressed inflammasome formation [14].

In the current study, we have evaluated the protective potential of TUDCA against oxidative damage and endoplasmic reticulum (ER) stress in human RPE cells. We found that TUDCA attenuated H_2_O_2_-evoked oxidative damage and inhibited thapsigargin (TG)-induced ER stress. 

## 2. Materials and Methods

### 2.1. Cell Viability

ARPE-19 cells (ATCC^®^ CRL-2302™) were cultured in T25 flasks with DMEM/F12 medium for 24 h in a 5% CO_2_ incubator at 37 °C. The cells were detached and seeded in 96-well plates (5 × 10^4^ cells/well) for 24 h then treated with H_2_O_2_, TG, TUDCA, H_2_O_2_ plus TUDCA, or TG plus TUDCA for 24 h. H_2_O_2_, at a concentration of 750 µM, was chosen for this study, based on our previous publication [15,16]; and 1 µM TG (Cat. T9033, Sigma, Dorset, UK) is commonly used to induce ER stress in vitro [17,18,19]—so we also used a dose of 1 µM for this study. TUDCA (Cat. 580549, Sigma, Dorset, UK) at different concentrations were used. Cell viability was evaluated using an MTT assay (Cat. M2128, Sigma, Dorset, UK) following the manufacturer’s protocol and percentage viability calculated according to our previous description [15]. 

### 2.2. Detection of Apoptosis

Cell death was detected using DeadEnd™ fluorometric terminal deoxynucleotidyl transferase dUTP nick end labeling (TUNEL) assay kit (Cat. G7360, Promega, Southampton, UK) according to the manufacturer’s guidance. Briefly, ARPE-19 cells were seeded on coverslips in DMEM/F12 medium in a 6-well plate (5 × 10^5^/well) for 24 h. The cells were subjected to treatment with H_2_O_2_ (750 µM) or H_2_O_2_ (750 µM) plus TUDCA (100 µM) for 24 h, then washed twice with phosphate-buffered saline (PBS) and fixed with 4% (*w*/*v*) paraformaldehyde for 25 min, followed by washing with PBS and permeabilizing with PBS containing 0.2% (*v*/*v*) Triton X-100. Cells were incubated with rTDT reaction mix, and the reaction was stopped with the saline-sodium citrate (SSC) *buffer* (2×). Cells were mounted with 4′,6-diamidino-2-phenylindole (DAPI) (Cat. D9542, Sigma, Dorset, UK) and FluorSave™ reagent (Cat. 345789, Merck Millipore, Watford, UK). Quantification of cell death was performed by counting the number of TUNEL positive cells using a ZEISS LSM 800 confocal microscope.

### 2.3. Quantification of Reactive Oxygen Species (ROS) Production

ARPE-19 cells (5 × 10^4^/well) were seeded in 96-well plates and incubated for 24 h. ARPE-19 cells were exposed to H_2_O_2_ (750 µM) or H_2_O_2_ (750 µM) plus TUDCA (100 µM) for 24 h. 6-Carboxy-20, 70-Dichlorofluorescin diacetate (DCFH-DA) (Cat. 4091-99-0, Sigma, Dorset, UK) was used to detect total ROS as described previously [15]. 

### 2.4. Quantitative Real-time Polymerase Chain Reaction (qRT-PCR)

ARPE-19 cells were seeded in DMEM/F12 medium in a 6-well plate (5 × 10^5^/well) for 24 h then incubated with H_2_O_2_ (750 µM) or H_2_O_2_ (750 µM) plus TUDCA (100 µM) for 24 h. The media were removed, and the cells were washed with PBS twice. Total RNA was extracted using Trizol Reagent (Cat. 93289, Sigma, Dorset, UK), and cDNA was synthesized using High-Capacity cDNA Reverse Transcription Kit (Cat. 4368814, Thermo Fisher Scientific, Paisley, UK), following manufacturers’ protocols. Targeted gene expression was detected using a Platinum^®^ SYBR^®^ Green QPCR SuperMix-UDG w/ROX kit following manufacturer guidelines (Cat. 4309155, Thermo Fisher Scientific, Paisley, UK). The relative gene expression was determined by a 2^−ΔΔCT^ formula. The primers for qRT-PCR are listed in Supplementary Table S1.

### 2.5. Biochemical Assays

ARPE-19 cells were seeded in DMEM/F12 medium in a 6-well plate (5 × 10^5^/well) for 24 h and then treated with H_2_O_2_ (750 µM) or H_2_O_2_ (750 µM) plus TUDCA (100 µM) for 24 h. The medium was discarded, the cells were scraped and homogenized in cold PBS. The cell lysates were centrifuged for 10 min at 12000× *g*, and the supernatants were collected. Catalase (CAT) and superoxide dismutase (SOD) activities were measured using, respectively, the OxiSelect Catalase Activity Assay Kit (Cat. STA-341, Cell Biolabs, San Diego, CA, USA) and the OxiSelect Superoxide Dismutase Activity Assay Kit (Cat. STA-340, Cell Biolabs, San Diego, CA, USA), according to the manufacturers’ instructions. Malondialdehyde (MDA) and Glutathione (GSH) levels were measured using, respectively, the TBARS assay kit (Cat. STA-330, Cell Biolabs, San Diego, CA, USA) and GSSG/GSH assay kit (Cat. STA-312, Cell Biolabs, San Diego, CA, USA), following the manufacturers’ guidelines.

### 2.6. Measurement of Caspase-3/7 Activities

ARPE-19 cells (5 × 10^4^/well) were cultured in a 96-well plate for 24 h and then exposed to H_2_O_2_ (750 µM) or H_2_O_2_ (750 µM) plus TUDCA (100 µM) overnight. Caspase-3 and -7 activities were measured with a Caspase-Glo 3/7 assay kit (Cat. G8090, Promega, Southampton, UK) following the manufacturer’s guidelines. 

### 2.7. Enzyme-linked Immunosorbent Assay (ELISA)

ARPE-19 cells were seeded in DMEM/F12 medium in a 6-well plate (5 × 10^5^/well) for 24 h, then subjected to treatment with H_2_O_2_ (750 µM) or H_2_O_2_ (750 µM) plus TUDCA (100 µM) for 24 h. The culture media were collected. Human Interleukin 1 beta (IL-1β) Mini ABTS ELISA Development Kit (Cat. 900-M95), IL-6 Mini ABTS ELISA Development Kit (Cat. 900-M16), and *tumor necrosis factor alpha* (TNF-α) Mini ABTS ELISA Development Kit (Cat. 900-M25), all purchased from PeproTech, London, UK, were used to measure, respectively, IL-1β, IL-6, and TNF-α, based on the manufacturer’s protocols. 

### 2.8. Statistical Analysis

For statistical analysis, data were analyzed using one-way Anova followed by Bonferroni post-hoc test (GraphPad Prism 6 software, version 6.0, GarphPad Software Inc., San Diego, CA, USA). All data were obtained after at least three independent experiments.

## 3. Results

### 3.1. Effects of H_2_O_2_ and TUDCA on Cell Viability

**Figure 1:**
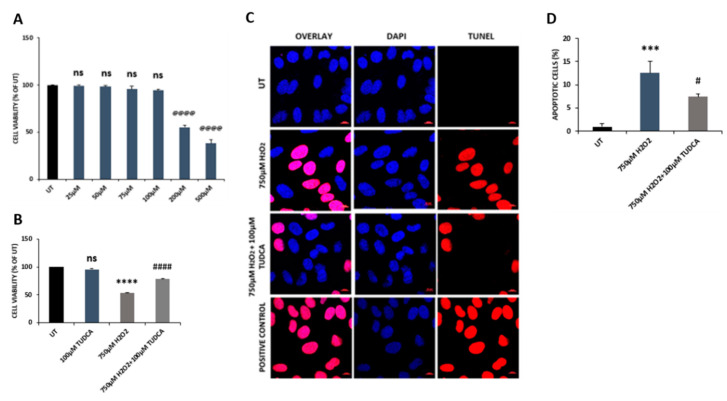
Tauroursodeoxycholic acid (TUDCA) counteracted H_2_O_2_-induced decrease in cell viability and increased in apoptotic cell death. (**A**) Cells were treated with TUDCA at different concentrations; higher concentrations (200 and 500 µM) caused significantly decreased cell viability, compared to the untreated (UT) control. (**B**) H_2_O_2_ treatment led to markedly decreased cell viability relative to control, while co-treatment with TUDCA significantly increased cell viability relative to H_2_O_2_ treatment alone. (**C**) Cell death was detected by the DeadEnd™ fluorometric terminal deoxynucleotidyl transferase dUTP nick end labeling (TUNEL) assay in untreated (UT), H_2_O_2_, and H_2_O_2_-plus-TUDCA-treated cells. Cells treated with DNase I (3000 U/mL) for 10 min were used as positive controls while the recombinant terminal deoxynucleotidyl transferase (rTdT) enzyme was excluded in the negative control subjects from the TUNEL reaction mixture. Nuclei were labeled with 4′,6-diamidino-2-phenylindole (DAPI) (scale bar, 10 µm) (**D**) Quantification of dead cells in control and treated cells. Data are shown as mean ± SEM. @@@@ *p* < 0.0001, TUDCA (200, 500 µM) vs. untreated (UT) control; *** *p* < 0.001, **** *p* < 0.0001, 750 µM H_2_O_2_ vs. UT; ^#^ *p* < 0.05, ^####^ *p* < 0.0001, 750 µM H_2_O_2_ plus TUDCA vs. 750 µM H_2_O_2_; ns, no significance, TUDCA (25, 50, 75, 100 µM) vs. UT.

**Figure 2:**
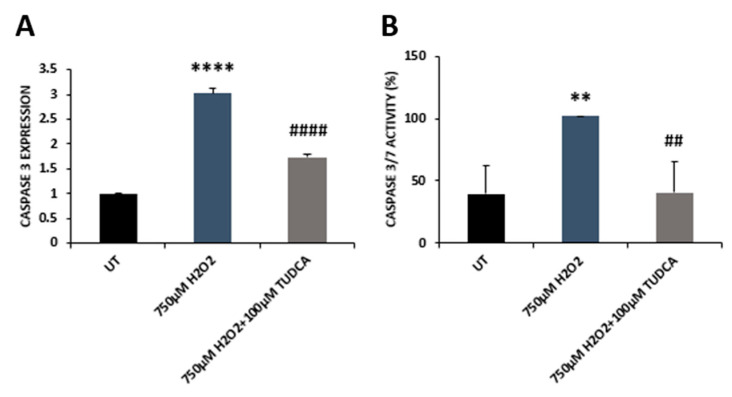
TUDCA decreases oxidative stress-induced caspase 3 expression and caspase 3/7 activity in ARPE-19 cells. (**A**) Caspase 3 mRNA in control (UT), H_2_O_2_, and H_2_O_2_-plus-TUDCA-treated cells were measured by qRT-PCR. (**B**) Activities of caspase 3 and 7 in control (UT), H_2_O_2,_ and H_2_O_2_-plus-TUDCA-treated cells were measured using CASPASE-Glo 3/7 assay. Data were presented as mean ± SEM. ** *p* < 0.01 and **** *p* < 0.0001, 750 µM H_2_O_2_ vs. untreated (UT) control; ^##^ *p* < 0.01, ^####^ *p* < 0.0001, 750 µM H_2_O_2_ plus TUDCA vs. 750 µM H_2_O_2_.

To examine the effect of TUDCA on cell viability, we exposed ARPE-19 cells to TUDCA at different concentrations (25, 50, 75, 100, 200, and 500 µM) and found that doses of 25, 50, 75, and 100 µM caused no difference in cell viability between control and treated cells. However, treatment with TUDCA at concentrations of 200 or 500 µM resulted in markedly decreased cell viability compared to that of control cells (Figure 1A). Consequently, TUDCA at 100 µM was used in further experiments. When cells were treated with H_2_O_2_ (750 µM) or H_2_O_2_ (750 µM) plus TUDCA (100 µM), similar to our previous findings of H_2_O_2_-induced effects [15,16], 750 µM H_2_O_2_ caused a marked decrease in cell viability compared to control cells, while co-treatment with H_2_O_2_ and TUDCA significantly increased cell viability compared to cells exposed to H_2_O_2_ alone (Figure 1B). To examine whether decreased cell viability was due to cell death, TUNEL assay was applied to detect apoptotic cells in control, H_2_O_2_, and H_2_O_2_-plus-TUDCA-treated cells. Cells incubated with H_2_O_2_ had a significant increase in cell death compared to control cells, while cells co-treated with H_2_O_2_ and TUDCA exhibited significantly decreased cell death compared to cells exposed to H_2_O_2_ alone (Figure 1C,D). In addition, we examined caspase 3 expressions and caspase 3 and 7 activities. H_2_O_2_ treatment upregulated caspase expression and increased activities of caspase 3 and 7 compared to control cells, while co-treatment with TUDCA significantly lowered caspase expression and activities of caspase 3 and 7 compared to cells exposed to H_2_O_2_ alone (Figure 2).

### 3.2. TUDCA Attenuated H_2_O_2_-induced Oxidative Stress in RPE Cells

**Figure 3:**
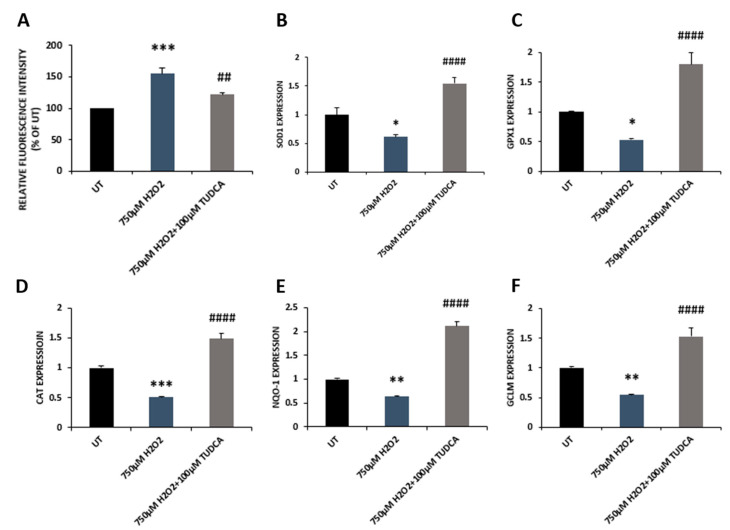
TUDCA reversed effects of H_2_O_2_ on ROS production and antioxidant gene expression. (**A**) ROS generation in control, H_2_O_2_, and H_2_O_2_-plus-TUDCA-treated cells was measured using DCFH-DA. (**B**–**F**) qRT-PCR was used to measure expression of antioxidant genes, *superoxide dismutase 1* (*SOD1*), *glutathione peroxidase 1* (*GPX1*), *Catalase* (CAT), *NAD(P)H dehydrogenase (quinone) 1* (*NQO-1*) and *glutamate-cysteine ligase modifier subunit* (*GCLM*). Data were shown as mean ± SEM. * *p* < 0.05, ** *p* < 0.01, *** *p* < 0.001, 750 µM H_2_O_2_ vs. untreated (UT) control; ^##^ *p* < 0.01, ^####^ *p* < 0.0001, 750 µM H_2_O_2_ plus TUDCA vs. 750 µM H_2_O_2_.

**Figure 4:**
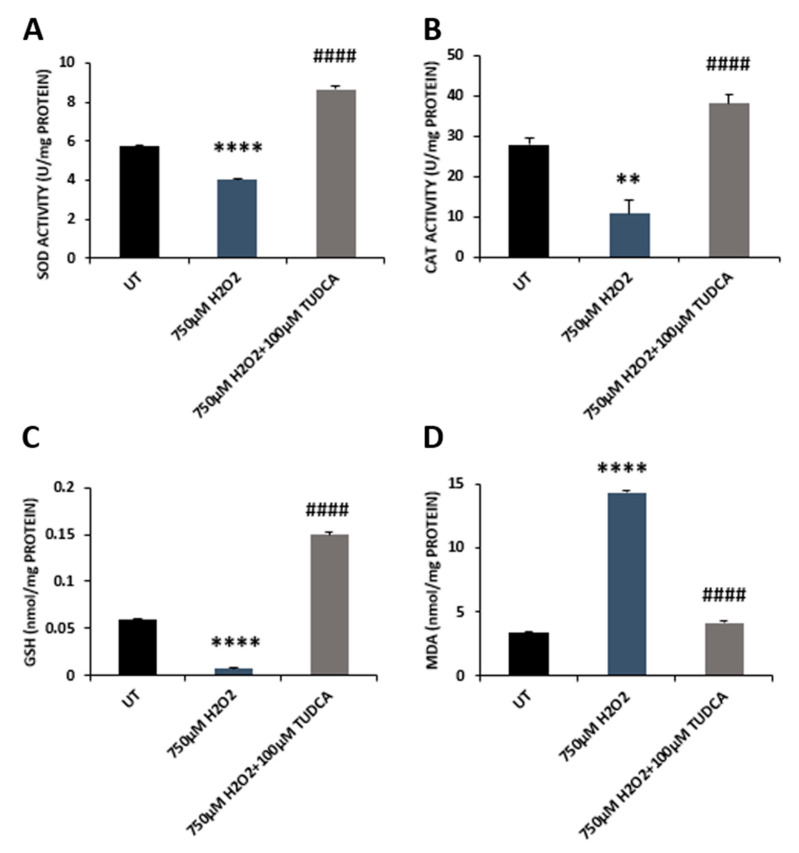
TUDCA counteracted H_2_O_2_-induced effects on activities of superoxide dismutase (SOD) (**A**) and catalase (**B**) and levels of glutathione (GSH) (**C**) and malondialdehyde (MDA) (**D**). Data are shown as mean ± SEM. CAT, catalase; UT, untreated (UT) control cells. ** *p* < 0.01, **** *p* < 0.0001, 750 µM H_2_O_2_ vs. UT; ^####^ *p* < 0.0001, 750 µM H_2_O_2_ plus TUDCA vs. 750 µM H_2_O_2_.

Our previous work showed that exposure of RPE cells to H_2_O_2_ resulted in oxidative stress [15,16]. In the current study, we examined whether TUDCA could reduce the H_2_O_2_-induced ROS production in ARPE-19 cells. DCFH-DA was used to determine total intracellular ROS, and the results showed that H_2_O_2_ treated cells had a markedly high level of ROS compared to control cells; co-treatment with TUDCA resulted in a significant decrease in ROS production compared to cells incubated with H_2_O_2_ only (Figure 3A). We further investigated intracellular antioxidant capacity in control, H_2_O_2_, and H_2_O_2_-plus-TUDCA-treated cells by measuring antioxidant gene expression. qRT-PCR data showed that cells treated with H_2_O_2_ had significantly reduced expression of antioxidant genes compared to that of control cells; co-treatment with TUDCA significantly increased the expression of these antioxidant genes compared to cells exposed to H_2_O_2_ alone (Figure 3B–F). Additionally, we evaluated the effects of TUDCA on SOD and CAT activities. H_2_O_2_ incubation significantly lowered SOD and CAT activities compared to control cells; co-incubation with TUDCA significantly increased SOD and CAT activities compared to cells treated with H_2_O_2_ alone (Figure 4A,B).

Glutathione (GSH) plays an important role in reducing the oxidative damage in cells. We measured GSH levels in control, H_2_O_2_, and H_2_O_2_-plus-TUDCA-treated cells and found that GSH level was significantly decreased in H_2_O_2_-treated cells compared to control cells; the level of GSH in co-treated cells was significantly increased compared to cells treated with H_2_O_2_ only (Figure 4C). We also examined the level of malondialdehyde (MDA), a marker for oxidative stress, and found that cells incubated with H_2_O_2_ showed a significant increase in MDA compared to control cells; cells co-incubated with TUDCA exhibited a significant decrease in MDA compared to cells treated with H_2_O_2_ alone (Figure 4D).

The nuclear factor erythroid 2–related factor 2 (NRF2) plays an important defensive role against oxidative stress by upregulating antioxidant gene expression [20]. We used qRT-PCR to measure NRF2 in control and treated cells. Cells exposed to H_2_O_2_ had a significant decrease in NRF2 compared to control cells; in cells treated with TUDCA plus H_2_O_2,_ NRF2 was significantly increased compared to that of cells treated with H_2_O_2_ only (Figure S1). 

### 3.3. TUDCA Inhibited H_2_O_2_-Induced Expression of Proinflammatory Cytokines in RPE Cells 

**Figure 5:**
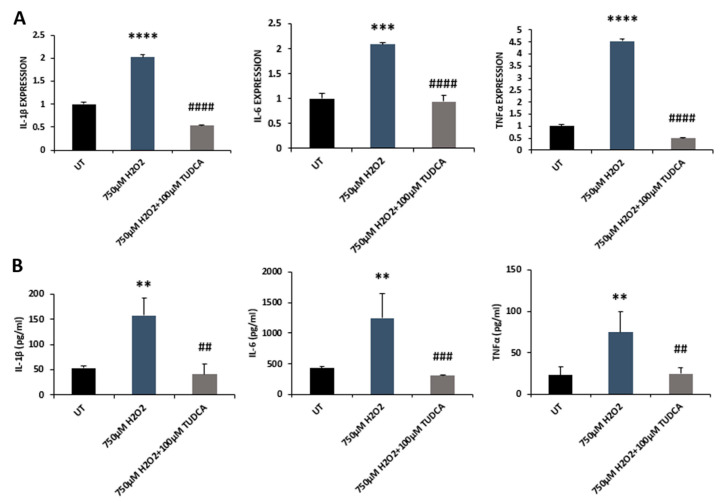
TUDCA suppressed H_2_O_2_-induced inflammation. (**A**) IL-1β, IL-6, and TNF-α mRNA levels in control, H_2_O_2_, and H_2_O_2_-plus-TUDCA-treated cells were measured by qRT-PCR. (**B**) Protein concentrations of IL-1β, IL-6, and TNF-α in control, H_2_O_2_, and H_2_O_2_-plus-TUDCA-treated cells were detected by ELISA. Data were shown as mean ± SEM. ** *p* < 0.01, *** *p* < 0.001, **** *p* < 0.0001, 750 µM H_2_O_2_ vs. untreated (UT) control; ^##^ *p* < 0.01, ^###^ *p* < 0.001, ^####^ *p* < 0.0001, 750 µM H_2_O_2_ plus TUDCA vs. 750 µM H_2_O_2_.

Inflammation has been implicated in the pathogenesis of inherited and complex retinal diseases [21]. There is a strong interrelationship between oxidative stress and inflammation [22]. We examined mRNA levels of IL-1β, IL-6, and TNF-α by qRT-PCR and showed that H_2_O_2_ exposure significantly increased IL-1β, IL-6, and TNF-α expression compared to that of control cells; co-exposure to TUDCA markedly lowered expression of IL-1β, Il-6 and TNF-α compared to cells exposed to H_2_O_2_ alone (Figure 5A). We also detected IL-1β, IL-6, and TNF-α protein by ELISA. The three cytokines were significantly increased in H_2_O_2_-treated cells compared to control cells. However, co-treatment with TUDCA significantly counteracted these H_2_O_2_-induced changes (Figure 5B).

### 3.4. TUDCA Attenuated Thapsigargin-Induced ER Stress in RPE Cells

**Figure 6:**
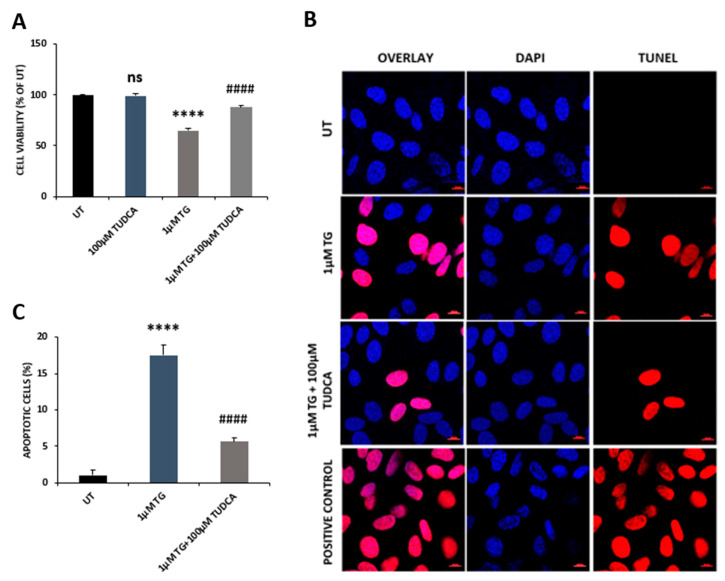
TUDCA counteracted the effects of thapsigargin (TG, 1µM) on ARPE-19 cell viability and apoptotic cell death. (**A**) Cell viability was examined using an MTT assay. (**B**) Apoptotic cells in control, TG, and TG-plus-TUDCA-treated cells were detected by TUNEL assay; nuclei were labeled with DAPI (scale bar, 10 µm). (**C**) The apoptotic cell rate in untreated (UT) control and treated cells was quantified. Data are shown as mean ± SEM. Ns, no significance, TUDCA vs. UT; **** *p* < 0.0001, 1 µM TG vs. UT; ^####^ *p* < 0.0001, 1 µM TG plus TUDCA vs. 1 µM TG.

**Figure 7:**
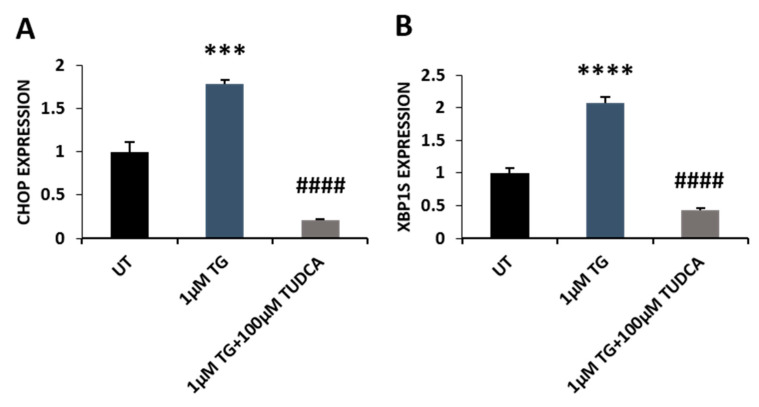
TUDCA suppressed TG-induced expression of ER sensors. (**A**) CHOP expression in untreated (UT) control, TG, and TG-plus-TUDCA-treated cells was examined by qRT-PCR. (**B**) Spliced XBP1 in untreated and treated cells was detected by qRT-PCR. Values were mean ± SEM. *** *p* < 0.001, **** *p* < 0.0001, 1 µM TG vs. UT; ^####^ *p* < 0.0001, 1 µM TG plus TUDCA vs. 1 µM TG.

ER stress has been widely considered to be associated with the pathogenesis of retinal degeneration [11]. We examined whether TUDCA suppresses ER stress in RPE cells. We incubated ARPE-19 cells with a widely used ER stress inducer, thapsigargin (TG, 1 µM), or TG plus TUDCA. Consistent with reports involving other cell lines [19], TG treatment caused a marked decrease in cell viability compared to that of control cells, while TUDCA counteracted this detrimental change (Figure 6A). ER stress can induce apoptotic cell death [11], so we used TUNEL assay to detect apoptotic cells in control, TG, and TG plus TUDCA treated cells. The number of apoptotic cells was markedly increased in TG-treated cells compared to control cells, while the number was significantly decreased in cells exposed to TG-plus-TUDCA compared to cells exposed to TG alone (Figure 6B,C). CCAAT-enhancer-binding protein homologous protein (CHOP) plays a critical role in mediating ER stress-induced apoptotic cell death. Being a transcription factor, CHOP can directly upregulate tribbles homolog 3 (TRB3) expression and subsequently inhibit protein kinase B activation, leading to apoptosis [23,24]. We measured CHOP expression in control, TG, and TG-plus-TUDCA-treated cells by qRT-PCR. CHOP expression was significantly increased in TG-treated cells at the mRNA level compared to control cells. TUDCA co-treatment significantly decreased CHOP expression compared to cells treated with TG only (Figure 7A). Activated IRE1 can induce XBP1 splicing and produce spliced XBP1, which subsequently upregulates Bip expression, and therefore, is also an indicator of ER stress [11,25]. We found that Spliced XBP1 was significantly increased in TG-treated cells compared to that of untreated cells, while it was markedly decreased in TUDCA plus TG exposed cells compared to cells exposed to TG alone (Figure 7B).

## 4. Discussion

Oxidative and ER stress plays a critical role in the progression of inherited and complex retinal degeneration, e.g., retinitis pigmentosa and AMD [2,11,26]. Suppression of oxidative and ER stress represents a promising therapeutic strategy for the treatment of retinal degeneration. As such, it would be of clear benefit to developing products that protect against oxidative and ER stress. TUDCA is one such product that is known to offer protection against retinal degeneration [6]. However, the underlying protective mechanisms are not well elucidated. Our current study demonstrated that TUDCA attenuated cell death, decreased ROS production, upregulated antioxidant gene expression, and inhibited inflammation in H_2_O_2_-treated RPE cells. TUDCA treatment also suppressed TG-induced ER stress and associated cell death.

TUDCA can prevent apoptotic cell death via inhibiting Bax translocation, cytochrome c release, and caspase activation [27]. Previous studies have shown that TUDCA inhibits retinal cell death by counteracting oxidative stress [28]. Gaspar et al. (2013) reported that TUDCA attenuated retinal neural cell death and decreased ROS production and protein oxidation induced by a high glucose concentration (30 mM) [29]. In a retinal detachment rat model, TUDCA prevented photoreceptor degeneration via decreasing oxidative stress and caspase activities [30]. Intense light can cause oxidative damage, resulting in photoreceptor degeneration. TUDCA treatment significantly reduced superoxide radicals, decreased photoreceptor death, and increased visual function in the retinas of mice treated with intense light [3,9]. A recent study reported that TUDCA preserved cone photoreceptors and visual function in n-methyl-n-nitrosourea-induced mouse retinal degeneration by suppressing the expression of apoptotic factors (caspase 3, calpain-2, and Bax), increasing SOD protein level, and reducing MDA formation [31]. In our current study, the data demonstrated that TUDCA also alleviated H_2_O_2_-induced oxidative damage in RPE cells. TUDCA treatment prevented caspase 3-dependent RPE apoptosis, indicated by a decrease in both caspase 3 expression and caspase 3/7 activities (Figure 1C,D, and Figure 2). It is possible, of course, that TUDCA might attenuate other H_2_O_2_-induced cell death pathways that were not examined. TUDCA treatment also enhanced antioxidant capacity in RPE cells exposed to H_2_O_2_ by upregulating antioxidant gene expression, increasing antioxidant enzyme activities and GSH level, and inhibiting MDA production (Figure 3 and Figure 4). Transcription factor NRF2, is a key regulator for antioxidant gene expression and plays a beneficial role in retinal cell protection against oxidative damage [2]. Loss of NRF2 in mice causes age-related RPE atrophy, choroidal neovascularization, and sub-RPE deposition [32]. Early studies demonstrated that TUDCA suppressed rifampicin-induced damage in HepG2 cells via upregulating expression of NRF2 at mRNA and protein levels [33]. TUDCA also increased NRF2 protein level in the midbrain and striatum of mice challenged with 1-methyl-4-phenyl-1,2,3,6-tetrahydropyridine (MPTP); subsequently, expression and activities of antioxidant enzymes, the downstream targets of NRF2, were also increased in MPTP-injected mouse midbrain and striatum with TUDCA treatment [34]. Our current work showed that TUDCA upregulated NRF2 expression at the mRNA level in RPE cells (Figure S1) and also increased the expression and activities of NRF2 downstream target antioxidant enzymes (Figure 3B–F and Figure 4A,B). 

Inflammation plays a key role in the progression of retinal degeneration [22]. Protection by TUDCA against inflammation has been shown in animals with different types of diseases, such as hepatic ischemia-reperfusion and acute neuroinflammation [28]. TUDCA has been demonstrated to have anti-inflammatory activity in the retinas of streptozotocin-induced diabetic rats by suppressing the expression of intercellular adhesion molecule 1 (ICAM-1), nitric oxide synthase (NOS), nuclear factor kappa-light-chain-enhancer of activated B cells (NF-κB) P65, and vascular endothelial growth factor (VEGF) [35]. Choroidal neovascularization (CNV) is one of the clinical features of AMD, the commonest cause of visual impairment in the aged population [2]. Laser-induced CNV formation in rats was suppressed by TUDCA treatment through its anti-inflammatory action [36]. In inherited retinal degeneration (retinitis pigmentosa, RP), TUDCA prevented photoreceptor degeneration in *rpgr* knockout mice, an RP mouse model. TUDCA also ameliorated microglial activation and infiltration to the photoreceptor layer [14]. Activated microglia can produce proinflammatory cytokines, e.g., IL-1β and TNF-α, and TUDCA treatment suppressed inflammasome formation and decreased IL-1β production in the *rpgr* knockout mouse retinas [14]. TUDCA also preserves retinal function in RHO^P23H/P23H^ transgenic rats, another RP model, through the inhibition of microglial activation [8,37]. Our current study also showed that TUDCA alleviated H_2_O_2_-induced inflammation in RPE cells, decreasing expression and secretion of proinflammatory cytokines, IL-1β, IL-6, and TNF-α (Figure 5), which may help to maintain RPE function and benefit photoreceptor cells. In fact, a previous study has shown that TUDCA reverses H_2_O_2_-induced phagocytosis impairment in RPE cells by activating the MerTK pathway [38], which may contribute to the prevention of photoreceptor degeneration under stress condition. 

ER stress is caused by an abnormal build-up of unfolded and misfolded proteins in the ER, which results in the activation of the unfolded protein response (UPR). UPR is mediated by the PERK, IRE1, and ATF6 signal pathways. When these pathways are activated, biosynthesis of unfolded or misfolded proteins is decreased, biosynthesis of chaperones is increased, and proteasome-mediated protein degradation is activated. Under severe ER stress, UPR activates an apoptotic process by upregulating the expression of CHOP [11,28]. TUDCA has been shown to exert chaperoning activity by promoting protein trafficking and increasing protein folding capacity via ATF6 activation. Abundant evidence has demonstrated that TUDCA alleviates ER stress in different cell types and disease animal models [27]. TUDCA also can inhibit ER stress-associated apoptosis through modulating intracellular calcium levels and blocking the activation of calpain and caspase-12 [27]. Mutation in the cyclic nucleotide-gated channel alpha subunit (*CNGA3*) gene causes achromatopsia 3 with cone dysfunction [38]. Overexpressed CNGA3 mutant proteins (CNGA3^R563H^ and CNGA3^Q655X^) were no longer localized to the plasma membrane and were retained in the ER, causing ER stress in photoreceptor 661W cells with higher XBP1 splicing and expression of Bip and CHOP; TUDCA treatment alleviated CNGA3 mutant protein-associated ER stress, enhanced the mutant protein’s cell surface localization and reduced ER retention [39]. TUDCA also preserved cone photoreceptor function in *lrat^-/-^* mice, a model for Leber congenital amaurosis (LCA) and early retinal dystrophy, through the reduction of ER stress and apoptosis. Mice treated with TUDCA had decreased CHOP protein and caspase activities in their retinas [40]. Our current data also demonstrated that TUDCA attenuated TG-induced ER stress and apoptosis in RPE cells, in which CHOP and spliced XBP1 expression was significantly reduced (Figure 6 and Figure 7). 

Our observation here was based on an in vitro non-polarized RPE cell model, which might not be exactly relevant to an in vivo study. Ideally, we can further examine the protection of TUDCA against oxidative and ER stress in polarized ARPE-19 and primary RPE cells and in preclinical models to fully verify the observation made. In addition, it would be important to gain more insights into the molecular mechanism of TUDCA’s protection *in vivo*. 

## 5. Conclusions

Our work demonstrates that TUDCA can protect RPE from oxidative stress, inflammation, and ER stress, and further supports that TUDCA has therapeutic potential for treating retinal degeneration. 

## Supplementary Materials

The following are available online at https://www.mdpi.com/2227-9059/8/9/367/s1, Figure S1: Protection of TUDCA against oxidative damage was mediated by NRF2. NRF expression in control, H_2_O_2_, and H_2_O_2_-plus-TUDCA-treated cells was measured by qRT-PCR, Table S1: Primers for qRT-PCT.
