# Supplementary material for: Tauroursodeoxycholic Acid Protects Retinal Pigment Epithelial Cells from Oxidative Injury and Endoplasmic Reticulum Stress In Vitro
Source: Biomedicines. 2020 Sep 21;8(9):367. doi: 10.3390/biomedicines8090367 (PMC7555559; doi:10.3390/biomedicines8090367)
Supplement: Supplementary file 1 [file biomedicines-08-00367-s001.pdf]

Supplementary data

Figure S1

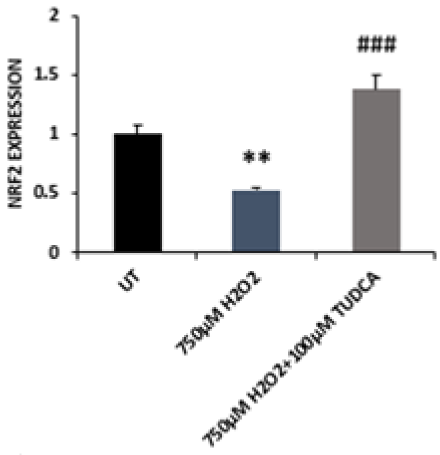

Figure S1 Protection of TUDCA against oxidative damage was mediated by NRF2. NRF expression in control, H_2O_2, and H_2O_2-plus-TUDCA-treated cells was measured by qRT-PCR.

Table S1 Primers for qRT-PCT

Genes	Forward primers 5′-3′	Reverse primers 5′-3′	PCR products (bp)
GPX 1	AGTCCACCGTGTATGCCTTC	CTCCTGGTGTCCGAACTGAT	218
SOD 1	AGGGCATCATCAATTTCGAG	CATTGCCCAAGTCTCCAAC	217
Catalase	ATCTCGTTGGAAATAACACC	AGAAACCTGATGCAGAGACT	161
NQO-1	CCTCTATGCCATGAACTT	TATAAGCCAGAACAGACTC	107
GCLM	GCCATAGGTACCTCTGATC	CTTGACAGACAACATACTGTC	487
NRF2	AGTGGATCTGCCAACTACTC	CATCTACAAACGGGAATGTCTG	106
Capase 3	AACTGGACTGTGGCATTG	ACCAGGTGCTGTGGAGTA	107
IL1-β	GCTGAGGAAGATGCTGGTTC	TCCATATCCTGTCCCTGGAG	213
IL-6	ATGTAGCCGCCCCACACAGA	CATCCATCTTTTTCAGCCAT	190
TNF-α	CCCTGAAAACAACCCTCAGA	CCACGATCAGGAAGGAGAAG	234
CHOP	CCAGCAGAGGTCACAAGCAC	GGGAATGACCACTCTGTTTC	126
XBP1S	GGGCTTGGTATATATGTGG	GGTCTGCTGAGTCCGCAGCAGG	156
β-ACTIN	TCCACGAAACTACCTTCAACTC	GTCATACTCCTGCTTGCTGAT	269
